# Radiation-Free Microwave Technology for Breast Lesion Detection Using Supervised Machine Learning Model

**DOI:** 10.3390/tomography9010010

**Published:** 2023-01-12

**Authors:** Soumya Prakash Rana, Maitreyee Dey, Riccardo Loretoni, Michele Duranti, Mohammad Ghavami, Sandra Dudley, Gianluigi Tiberi

**Affiliations:** 1 School of Engineering, London South Bank University, London SE1 0AA, UK; 2 Breast Screening and Diagnostic Breast Cancer Unit, AUSL Umbria 2, 06034 Foligno, Italy; 3 Department of Diagnostic Imaging, Perugia Hospital, 06156 Perugia, Italy; 4 Umbria Bioengineering Technologies (UBT) Srl, 06081 Perugia, Italy

**Keywords:** radiation-free technology, non-invasive lesion detection, X-ray free breast screening, MammoWave’s dielectric breast response, supervised machine learning

## Abstract

Mammography is the gold standard technology for breast screening, which has been demonstrated through different randomized controlled trials to reduce breast cancer mortality. However, mammography has limitations and potential harms, such as the use of ionizing radiation. To overcome the ionizing radiation exposure issues, a novel device (i.e. MammoWave) based on low-power radio-frequency signals has been developed for breast lesion detection. The MammoWave is a microwave device and is under clinical validation phase in several hospitals across Europe. The device transmits non-invasive microwave signals through the breast and accumulates the backscattered (returned) signatures, commonly denoted as the $S_{21}$ signals in engineering terminology. Backscattered (complex) $S_{21}$ signals exploit the contrast in dielectric properties of breasts with and without lesions. The proposed research is aimed to automatically segregate these two types of signal responses by applying appropriate supervised machine learning (ML) algorithm for the data emerging from this research. The support vector machine with radial basis function has been employed here. The proposed algorithm has been trained and tested using microwave breast response data collected at one of the clinical validation centres. Statistical evaluation indicates that the proposed ML model can recognise the MammoWave breasts signal with no radiological finding (NF) and with radiological findings (WF), i.e., may be the presence of benign or malignant lesions. A sensitivity of 84.40% and a specificity of 95.50% have been achieved in NF/WF recognition using the proposed ML model.

## 1. Introduction

Breast cancer is the most common cancer in women worldwide, affecting 1 in every 8 women [1]. Mammography is the gold standard technology for breast screening but it has ionizing radiation which leads to potential harms for patients; specifically, the cumulative effect of routine mammography screening may increase women’s risk of developing radiation-induced breast cancer [2]. Thus, age and screening frequency have been defined taking into account mammography risk-benefit ratio. Also, women feel some pain and discomfort [3] when undergoing mammography. Specifically, many women avoid mammography screening by fear of pain, embarrassment, discomfort, and radiation [1]. Additionally, the level of anxiety about the screening outcome is a tangible factor reducing mammography screening adherence, although the attitude differs depending on the age, profession, marital status, ethnicity, racial and educational differences [4,5].

To overcome the fear of pain, discomfort and ionizing radiation exposure issues, a novel device (i.e. MammoWave) based on low-power radio-frequency signals has been developed for breast lesion detection by UBT S.R.L. (Italy) team [6].

Moreover, in some cases, lesions prove difficult to detect from mammography, especially when the breast is highly dense [7,8,9], or if the breast comprises small, elongated salt-like microcalcification particles [10]. However, the evolution of machine learning (ML) algorithms and the greater availability of medical datasets from different modalities is enabling improved assisted detection and better performance hopes [11,12,13].

A recent case study on early breast cancer detection using AI methods from the mamographic images has been demonstrated in [14]. This study focus on the data collected from UK and US clinical trials. They shown a cancer case (small, irregular mass with associated microcalcifications and ) that was missed by six readers in the examination, but correctly identified by the AI system. In this case three level deep learning was used to train the model for the breast cancer detection. In [15], a background on the key ethical, technical, legal and regulatory challenges of AI in breast imaging and performance in breast screening have been provided.

Recently, microwave-based techniques have emerged and received attention as an alternative breast-screening tool [6,16,17,18,19]. Ultrawide band microwave breast imaging (UWB-MWBI) demonstrates strong evidence of the dielectric property contrast between healthy and malignant tissues. Usually, a multistatic or monostatic radar mechanism is employed to measure the dielectric property contrast of breast tissues in the spectrum between 0.5 GHz to 9 GHz [20,21]. Hitherto, seven clinically tested MWBI operational systems have been reported in the literature such as; (a) UBT Srl, Italy who designed a MWBI system, named MammoWave, differentiate healthy tissues and tissues with lesions. MammoWave has been tested on 150 patients [22]; (b) Dartmouth College, USA, constructed a MWBI device for breast cancer identification where patterns were inspected by a the Surgical Pathology team at Dartmouth-Hitchcock Medical Center (DHMC). The trial was performed with 150 patients with and without lesions [23]; (c) Multistatic Array Processing for Radiowave Image Acquisition (MARIA) system was developed at the University of Bristol, UK and a clinical trial was carried with over 300 patients including healthy and lesion breast patterns [24,25]; (d) Tissue Sensing Adaptive Radar (TSAR) system was developed at the University of Calgary, CA and a clinical trial was reported with a small group of patients [26,27]; (e) Southern University of Science and Technology, China was constructed a MWBI imaging system and completed their first trial with a small group of patients [28]; ( f ) Hiroshima University, Japan trialled a MWBI for cancer identification with a small group of patients [29]. Additionally, McGill University, Canada [30] and Shizuoka University, Japan [31] have also developed their own MWBI system and recently started trials.

Microwave breast screening is non-ionising, non-invasive, and painless as it does not include any form of breast compression. A handful of microwave-based studies have been performed by researchers to detect breast cancer using real data, with the majority investigated using either numerical simulations or with phantoms. UBT Srl’s MammoWave is one of the few Ultra-Wide Band (UWB) based microwave breast screening prototypes built, tested, and validated. MammoWave, uniquely functions in air with 2 antennas rotating in the azimuth plane, operating within the frequency band of 1–9 GHz. MammoWave examinations record the complex $S_{21}$ (backscattered (returned) complex signals), in the frequency domain. Artefact removal is performed through appropriate mathematical procedures, namely rotation subtraction [32,33].

### Contribution towards This Research

This work demonstrates that Machine learning (ML) can help understand phenomena from the frequency spectrum collected through MammoWave in response to the stimulus, segregating breasts with and without lesions. Specifically, ML can recognise the MammoWave signal response of breasts with no radiological finding (NF) and breasts with radiological findings (WF), i.e., with lesions (finding) which may be benign or malignant. Contributions of this study are: (a) the experiment has been performed across 61 breasts, enabling the exploration of lesions with different dimensions. (b) The new data appear differently in the hyperplane, motivating the authors to explore Gaussian kernel of SVM alongside of quadratic kernel ($SVM_{Q}$ ) and Gaussian kernel ($SVM_{G}$), which are found to be more efficient in this case. (c) Making better use of the frequency response signals has been explored and experimentally it is shown that 50 components obtained using principal component analysis (PCA) provide best classification in this case. (d) The prediction results have been analysed by the team of researchers and radiologists through statistical measurements to understand the false positive and negative classifications, revealing that lesion size and breast density have effect on microwave response as well as ML predictions.

## 2. Materials and Methods

**Figure 1:**
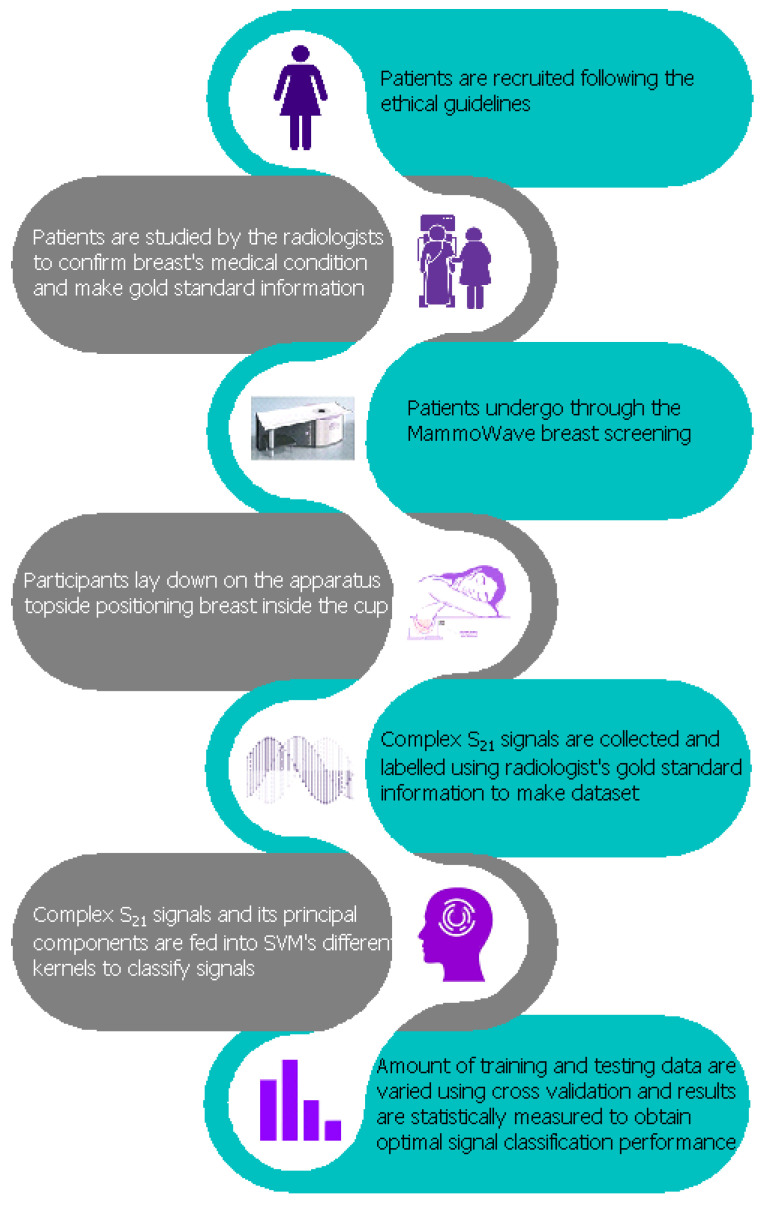
Proposed flow chart of MammoWave signal classification for the breast lesion detection.

A diagrammatic interpretation of the proposed work is presented in Figure 1. Each breast has its own correspondent output of the radiologist study assessment, which has been used as gold standard for categorization of the breasts in two categories: breasts with no radiological finding (NF), and breasts with radiological findings (WF), i.e., with lesions which may be benign or malignant.

Regarding the radiological study review, the radiologist assessment (NF/WF) used (accordingly to his/her opinion) one or more of the following conventional techniques: mammography, performed using Selenia LORAD Mammography System (Hologic, Marlborough, MA, USA); echography, performed using the MyLab 70 xvg Ultrasound Scanner (Esaote, Genova, Italy); magnetic resonance imaging, performed through a 3.0 T MAGNETOM scanner (Siemens Healthcare, Erlangen, Germany). Gold standard labels of the breasts (NF or WF) have been employed to train and test the ML algorithms to automatically identify breast response signals backscattered from lesions via the MammoWave. The approach follows six primary steps: patient’s undergo breast examinations through conventional approaches and breast type annotation (WF or NF) by the resident radiologist; subsequently, patient’s undergo breast examination through MammoWave and the microwave signal data is collected. Once this is done, classification of resulting microwave signals is performed. The reduction of microwave feature space applying PCA to improve the classification result then follows. Finally, classification of signals employing only the real fragments of the complex signals, reduction of real part’s feature space for improving the classification result. Each phase is explained in the following subsections.

The proposed research aims to identify the optimal classification settings for the research question using the components of complex $S_{21}$ numbers. There are four possible ways to employ complex numbers (not alter the form of the original signal)and perform the classification task: (i) applying actual complex $S_{21}$ responses, (ii) using features extracted from the complex $S_{21}$ responses, (iii) using resistance values (real part of the complex $S_{21}$ responses) from the transmitted voltage and current (reactance or imaginary part of $S_{21}$ varies inversely with increasing frequency thus unsuitable for this classification task), (iv) using features extracted from the real part of $S_{21}$. The possible classification directions involved in the proposed study to improve breast lesion identification performances detailed in the later section.

- MammoWave breast signal classification considering features extracted from the complex $S_{21}$ responses. Features have been extracted using PCA a powerful mathematical tool for multivariate data transformation. $SVM_{Q}$ has been chosen for the ML task applying the team’s previous research. Subsequently, $SVM_{G}$ has been experimented alongside observing spherical data shapes for improved classification $SVM_{G}$ performed better than $SVM_{Q}$ here, thus $SVM_{G}$ has been further adopted for the following classification tasks.
- MammoWave breast signal classification considering real parts of complex $S_{21}$ responses and employing $SVM_{G}$.
- MammoWave breast signal classification considering features extracted (by PCA) from real parts of complex $S_{21}$ and employing $SVM_{G}$.

Characteristics of the data used in each of these stages and their performance summaries are explained in the following sections.

### Apparatus Description and Data Collection

**Figure 2:**
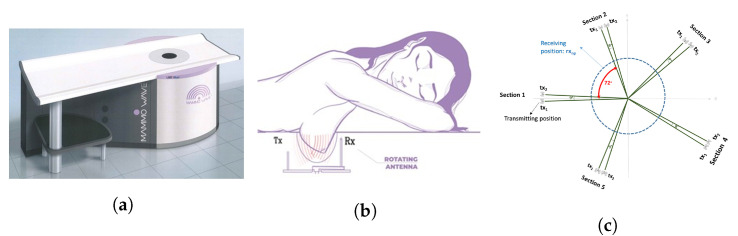
(**a**) MammoWave device designed by Umbria Bioengineering Technologies (UBT), Italy. (**b**) Patient’s breast examination procedure with MammoWave. (**c**) Pictorial view of the transmitting-receiving antenna positions of MammoWave measurement.

The MammoWave device (shown in Figure 2a) has been designed by Umbria Bioengineering Technologies (UBT), Italy. The device comprises a cylindrical aluminium container equipped with a chamber and cup to comfortably place the breast when women (or, participants) lay down to be screened. There is one transmitting ($t_{x}$) and one receiving antenna ($r_{x}$) which operate at 1 to 9 GHz frequency to obtain the breast’s response to the applied microwave signals. The 2 antennas rotate around the breast, illuminating it from a number of angles. Figure 2b displays how patients undergo the MammoWave’s breast examination, placing the breast inside the chamber with no breast compression required. To support all patients, three different cup sizes are available, and the best fit for the patient breast is selected. The largest cup has a diameter of 135 mm; thus, it cannot accommodate very large breasts. The cups have the following features. They are made using polylactic acid (PLA) to ensure biocompatibility [34] and with a width of 1 mm. This 1 mm thickness is based on modelling and experimental investigations by the team which demonstrated that this thickness has no effect on the microwave imaging outcomes [35].

The functioning principle of the MammoWave system is based on the dielectric properties (i.e., relative dielectric constant and conductivity) contrast between breast normal tissue and tissue with lesions at microwave frequencies. The antennas inside the container (covered to absorb microwaves) are fitted at the constant height, in free space and can rotate across the azimuth for collecting the microwave signals from diverse angular locations. The transmitting and receiving antennas are attached to a 2-port VNA (Cobalt C1209, Copper Mountain, Indianapolis, IN) that operates up to 9 GHz. Measurements have been accomplished by recording the complex $S_{21}$ in a multi-bistatic fashion. For every transmitting and receiving spot, the complex $S_{21}$ is gathered from 1 to 9 GHz, along with 5 MHz sampling. Let, $r_{x}$ rotate across the azimuth collecting the microwave signals from diverse angular at a radius $a_{0}$. The received signals can be expressed as, *n* = 1,2, …,80, denotes the receiving points; *m* = 1,2, …,5 indicates the transmitting sections, *p* = 1,2 indicates the position inside each transmitting section; and *f* is the frequency.

MammoWave uniquely functions in air with 2 antennas rotating in the azimuth plane, operating within the frequency band of 1–9 GHz. The 2 antennas, one transmitting and one receiving, both rotate around the breast, illuminating it from a number of angles. MammoWave examinations are performed by recording the $S_{21}$ (backscattered (returned) complex signals), in the frequency domain. Artefact removal is performed through appropriate mathematical procedures, namely rotation subtraction [32,33]. The MammoWave system has been employed to collect patient data: according to the conventional radiologist review, 25 breasts without lesions and 36 breasts with lesions, have been used in the work. Different $S_{21}$ signatures are found when the microwave signals interact through breast tissues. This signature is due to the contrast in dielectric properties i.e., permittivity and conductivity, within the spectrum of microwave frequencies as they pass through the breasts. A high contrast (up to 5) has been reported [36] between healthy breast tissue and malignant tissue, while recent studies confirm a high contrast only between fatty and malignant breast tissues, while it decreases between healthy fibro glandular and malignant tissues [37].

MammoWave removes the need for applying any matching liquid. For each breast, measurements have been performed recording the complex $S_{21}$ in a multibistatic fashion. Specifically, we employed here 15 transmitting positions, displaced in 5 triplets centred at $0^{\circ }$, $72^{\circ }$, $144^{\circ }$, $216^{\circ }$, and $288^{\circ }$; in each triplet, the transmitting positions are displaced by $4.5^{\circ }$. For each transmitting position txm, the receiving antenna is moved to measure the received signal at 80 receiving points $rx_{n}$, equally spaced along the azimuth of $4.5^{\circ }$. For each transmitting and receiving position, we recorded the complex $S_{21}$ in the frequency band from 1 to 9 GHz, using a frequency sampling of 5 MHz. Thus, that for each breast, the raw-data can be represented by a matrix of complex $S_{21}$ with dimension (15×80)×(1601×2). Figure 2c shows a pictorial view of the measurements setup. A breast scan is completed in ∼10 min, whilst the person is in a horizontal face down position on a thin mattress, with no breast compression. Numbers and displacements of transmitting and/or receiving positions can be changed [35]; specifically, an increase of the number of transmitting and/or receiving positions will lead to an increase of the measurement time. We verified in phantoms that the proposed configuration allows detection in a reasonable measurement time [35], i.e., ∼10 min, a duration which is similar to a traditional X-ray breast screening examination.

The MammoWave feasibility clinical validation has been performed in Perugia and Foligno Hospitals, Italy (Ethical Committee of Umbria, Italy, approval N. 6845/15/AV/DM of 14/10/2015, N. 10352/17/NCAV of 16/03/2017, N 13203/18/NCAV of 17/04/2018). The correspondent clinical protocol aims to quantify the device’s accuracy in breast lesions detection. As an inclusion criterion, the subjects should have a radiologist study output obtained through conventional examinations (mammography and/or ultrasound and/or magnetic resonance imaging) within the last month. The protocol and procedures were in accordance with institutional and ethical standards in research, with Declaration of Helsinki (1964) and its later amendments.

For this study, we used data of 61 breasts, each one with its own correspondent radiologist study review output, which has been used as gold standard for classifying the breasts in two categories: breasts with no radiological finding (NF), and breasts with radiological findings (WF), i.e., with lesions which may be benign or malignant. Some details of the detected or suspected lesions have been collected for WF breasts; moreover, pathology and/or clinical follow-up has been performed for lesions’ final assessment (benign/malignant). The subject’s information is charted in Table 1. In Table 2 some details of the radiologist study review are given for WF breasts.

## 3. Proposed Methodology

**Figure 3:**
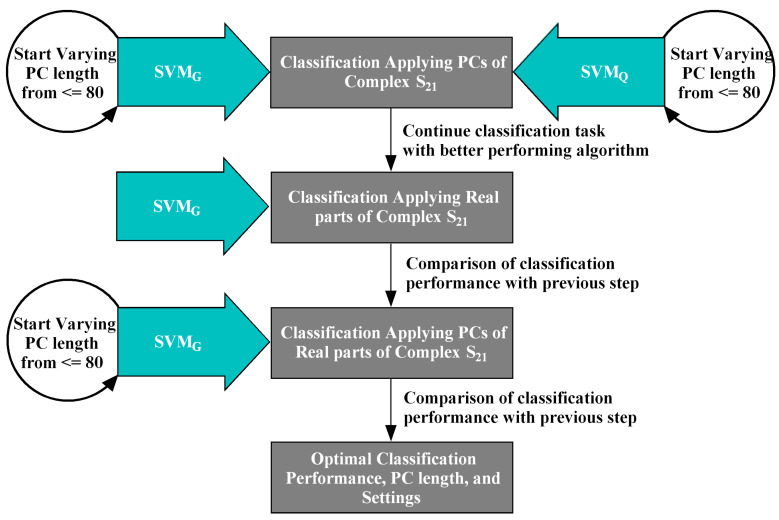
A Flowchart of the classification stages involved in the proposed ML experiment. $SVM_{G}$ and $SVM_{Q}$ have been trialled and compared, employing PCs of the complex $S_{21}$ data (raw data). The investigation continued with $SVM_{G}$, comparing its performance with $SVM_{Q}$. Subsequently, real parts and PCs obtained from the real parts of the complex $S_{21}$ have been employed in the second and third stages respectively to classify the NF-WF signals by $SVM_{G}$. The optimal performance attained in the third stage applying PCs of real parts of complex $S_{21}$ with $SVM_{G}$.

The proposed ML for segregating breasts with and without lesions from the MammoWave signal consist of mainly three stages. At first the PCA has been applied to the collected data from 61 breasts for the optimum use of the frequency response signals. Then SVM was applied on the extracted feature by PCA for the classification and results were compared with two different kernel functions. Finally, the performance of the classification method were analysed and results were validated statistically. A flow chart of the proposed method shown in Figure 3.

### 3.1. Principal Component Analysis

Principal components (PCs) have been extracted from the MammoWave’s complex $S_{21}$ responses, represented as $\lambda =\sum_{n=1}^{NF=1601}\lambda_{real}\left(n\right)+j\lambda_{img}\left(n\right)$, where *n* is the number of frequencies, $\lambda_{real}$ and $\lambda_{img}$ are the real and imaginary components respectively. The The $\lambda_{real}$ indicates the resistance of the dielectric materials (tissues) to the transmitted signals with *n* number of frequencies. The $\lambda_{img}$ indicates the capacitance of the dielectric materials (tissues) of the transmitted signals with *n* number of frequencies. PCA has been implemented on both $\lambda_{real}$ and $\lambda_{img}$ components to extract principal components (PCs) for classifying signals obtained from breast considering covariance matrix and Eigen vector.

### 3.2. Basic Theory of the Proposed Algorithms

Several ML algorithms present for classification in the literature, although the selection of appropriate methods is quite intuitive and needs to be determined heuristically. In support vector machine (SVM), each breast signal data consist of S− dimensional feature vector and a class is refer to each corresponding pattern [38]. SVM assigns a class label to each signal data pattern on the basis of its position with respect to a decision hyperplane, which defines by Equation (1), where, *b* is bias and *x* is variable. The distance between those boundaries of a margin area around the decision hyperplane, is called the margin width defined by $w^{T}x+b=\pm 1$. In SVM, kernel function must be chosen in order to achieve better performance. It defines the structure of the high-dimensional feature space to determine maximise margin hyperplane Equation (2).

(1) $$w^{T}x+b=0$$

(2) $$\hat{w},\hat{b}=\arg\min_{w,b}\left(\frac{1}{2}{∥w∥}^{2}\right)$$

The experimented data distribution appears non-linearly separable and to avoid model overfitting, hence the quadratic kernel ($SVM_{Q}$) and Gaussian kernel ($SVM_{G}$) function have been chosen for this NF and WF signal classification.

$SVM_{Q}$ have been employed to differentiate data points by minimising the gap between the two groups. The considered quadratic function is obtained by the optimisation problem define by Equation (3), where, $x_{i},x_{j}$ are real valued vectors, *d* is degree of polynomials, here d=2 (quadratic), since larger value tend to overfit.

(3) $$k\left(x_{i},x_{j}\right)={\left(x_{i}\cdot x_{j}+1\right)}^{d}$$

$SVM_{G}$ is popular kernel function for its excellent learning performance which can approximate bounded and continuous functions arbitrarily well, defined by Equation (4). The non-linear Euclidean distance controlled by kernel width parameter σ, which has great influence for the classification accuracy. It determine the feature space that the samples will be mapped onto. As σ→0, where all samples is classified correctly, but learning generalisation performance is poor and SVM can not classify new samples; whereas σ→∞, the whole sample is classified as one class. This function creates the best hyperplane to classify the subjects here.

(4) $$k\left(x_{i},x_{j}\right)=exp\left(-\frac{∥x_{i}-x_{j}{∥}^{2}}{2\sigma^{2}}\right),$$

### 3.3. Performance Analysis

In this work, 61 breasts were used, of which: 26 NF and 35 WF breasts (see Table 2). A summary statistic of two raw microwave scan population is shown in Table 3. Quantitative range of features of each population are not significantly high and compact in nature. Hence, application of normalisation could make the features insignificant for machine learning application and has been avoided in this work.

MammoWave’s breast screening data is non-linearly separable and logistic regression assumes the linearity between dependent and independent variables. Hence, logistic regression algorithm has not been attempted for classifying the data into WF and NF classes. In case of decision tree, a small change in the test/unseen data can cause a large change in the structure of the decision tree causing instability. Also, decision tree is very much sensitive to the new data which may put the whole system into risk. Therefore, decision tree has not been employed for the classification task. The leading supervised and non-linear classifiers such as, k-nearest neighbour, and multi-layer perceptron neural network, support vector machines have been attempted before and reported in the previously published article [39]. Support vector machine’s quadratic ($SVM_{Q}$) kernel has been selected following the previous research findings. As $SVM_{Q}$ was applied on a limited number of patients after performing dedicated $S_{21}$ pre-processing for artefact removal. Here, with the aim of exploring the applicability of ML algorithms directly to $S_{21}$, i.e., raw-data and observing spherical data shapes. Hence, the two algorithms $SVM_{G}$ and $SVM_{Q}$ are investigated and compared for obtaining improved classification performance. Also, the proposed work explores the possible way for improving previous results using principal component analysis (PCA) [40] on raw $S_{21}$ signals (described in Section 4.1), real parts of $S_{21}$ signals (described in Section 4.2, and PCA over real parts of $S_{21}$ signals (described in Section 4.3 minimising false positive-negative signals and identify the appropriate numerical sequence for the classification of NF and WF signals. It is possible that even the data are labelled into two groups by the radiologists but the finite differences are not suitable for classification when the elements of $S_{21}$ and principal components are prepared. Therefore, two sample *t*-test has been implemented three times before applying machine learning algorithm. Null hypothesis ($H_{0}$) of the proposed work assumes the $S_{21}$ values or extracted features applying PCA of two population (NF and WF group) comes from independent random samples from normal distributions with equal means and equal but unknown variances. However, the alternative hypothesis ($H_{a}$) assumes the $S_{21}$ values or extracted features applying PCA of two population (NF and WF group) comes from unequal means. Hence, $H_{a}$ needs to be accepted in order to perform NF-WF signal classification. The ideal importance level α=0.05 has been expected for tolerating and dismissing the null hypothesis, where *p*-value has been thought about for choosing the factual importance. Additionally, the confidence interval for the distinction in populace method for NF and WF signals have been considered, where $C_{L}$ and $C_{U}$ show the lower and upper limits of the certainty span. The ML experiment has been divided into two major parts; (a) realisation of optimal feature combination through training and validation phase (described in Section 4.1–Section 4.3), (b) testing the trained model with optimal settings (described in Section 4.4). The data have partitioned using Monte Carlo cross validation (MCCV) [41] in both cases. This is done because it randomly partitions to select the training and validation dataset helping to understand the impact of risk and uncertainty in NF-WF breast signal prediction. The performance outcomes (statistical metrics) have been aggregated and averaged over all the rounds. A number of statistical metrics, accuracy, sensitivity, and specificity have been used to investigate the classification performance of the classifiers [42]. Subsequently, Matthews Correlation Coefficient (MCC) [43] has been implemented to investigate the classification outcomes and estimate quality of classification and probability of the informed decision respectively. Receiver operating characteristic (ROC) curve has been generated for the optimally performing ML model to explain the diagnostic competence and steadiness of the classification system with different discrimination threshold. The hyperparameter optimisation has been conducted in training-validation phase and best operating point decided analysing ROC curve. While the optimised parameters and ROC threshold have been used to produce final testing result (described in Section 4.4).

## 4. Results Analysis

According to the radiologist’s review, a total of 34 patients have been included in this study, with a total of 61 breasts examined (see Table 1). Among the total examined breasts, 25 NF and 36 WF breasts underwent the MammoWave exam, collecting $S_{21}$ raw data. WF breasts are breasts having lesions, which may be benign or malignant; lesions were found to have dimensions ranging up to 32 mm. The raw-data of each breast are represented by a matrix of complex $S_{21}$ having dimensions 1200×3202 (described in Section 2), where each complex signal contains 1601 real and 1601 imaginary components. Hence the classification experiment has been conducted on total 73,200 signals (31,200 NF signals and 42,000 WF signals). Complex $S_{21}$ raw data signals and its components have been individually employed for experimental purpose, where prediction efficiency is influenced by the discriminating ability of individual features i.e., real, and imaginary parts of complex $S_{21}$. Each simulation has been run twenty-five times to observe the result stability before reporting average performance metrices.

### 4.1. Classification Applying PCs of Complex $S_{21}$

**Figure 4:**
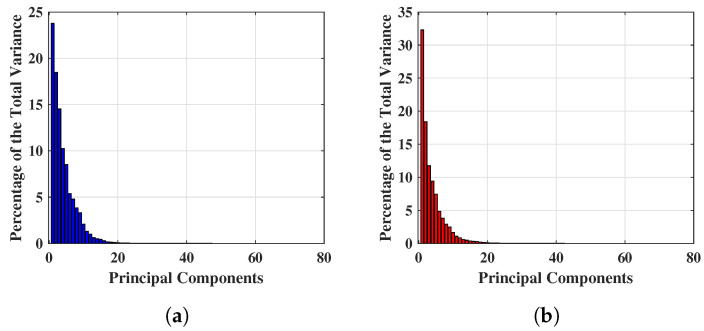
Example of significant variance achieved applying PCA on complex $S_{21}$ signals. (**a**) Significant variance achieved applying PCA on complex $S_{21}$ signals of a NF breast. (**b**) Significant variance achieved applying PCA on complex $S_{21}$ signals of a WF breast.

Figure 4 shows the percentage of total variance obtained from each PC for two different breast $S_{21}$’s, and the first 80 PCs are found to be quantitatively significant. This is because numerically, the variance values are a factor of 10${}^{-5}$ after the 80 PCs, hence extremely small and quantitatively insignificant, thus the team will investigate the first 80 PCs only. Figure 4a,b show an example of percentage variance for the first 80 PCs, where the x− axis and y− axis represent the number of components and percentage of variance respectively. Figure 4a displays the percentage of variance of a NF breast and Figure 4b describes the percentage of variance of a WF breast. Hence, the experiment for reduced feature space has been started from 80 PCs and features are continually eliminated until the optimal performance achieved with this feature setting. Maximum variances of 23.774% and 32.289% were achieved for NF and WF breast contained the within 80 PCs mentioned above.

Table 4 shows the outcomes of the *t*-test, where p<α rejects the null hypothesis $H_{0}$ ($H_{0}=1$), accepts alternative hypothesis $H_{a}$, and the true mean of the population belong between $-3\times 10^{-5}$ to $-1.500\times 10^{-5}$. Hence, the acceptance of alternative hypothesis indicates that the $\lambda_{real}$ data comes from populations with unequal means and can be employed for NF-WF signal classification task.

**Figure 5:**
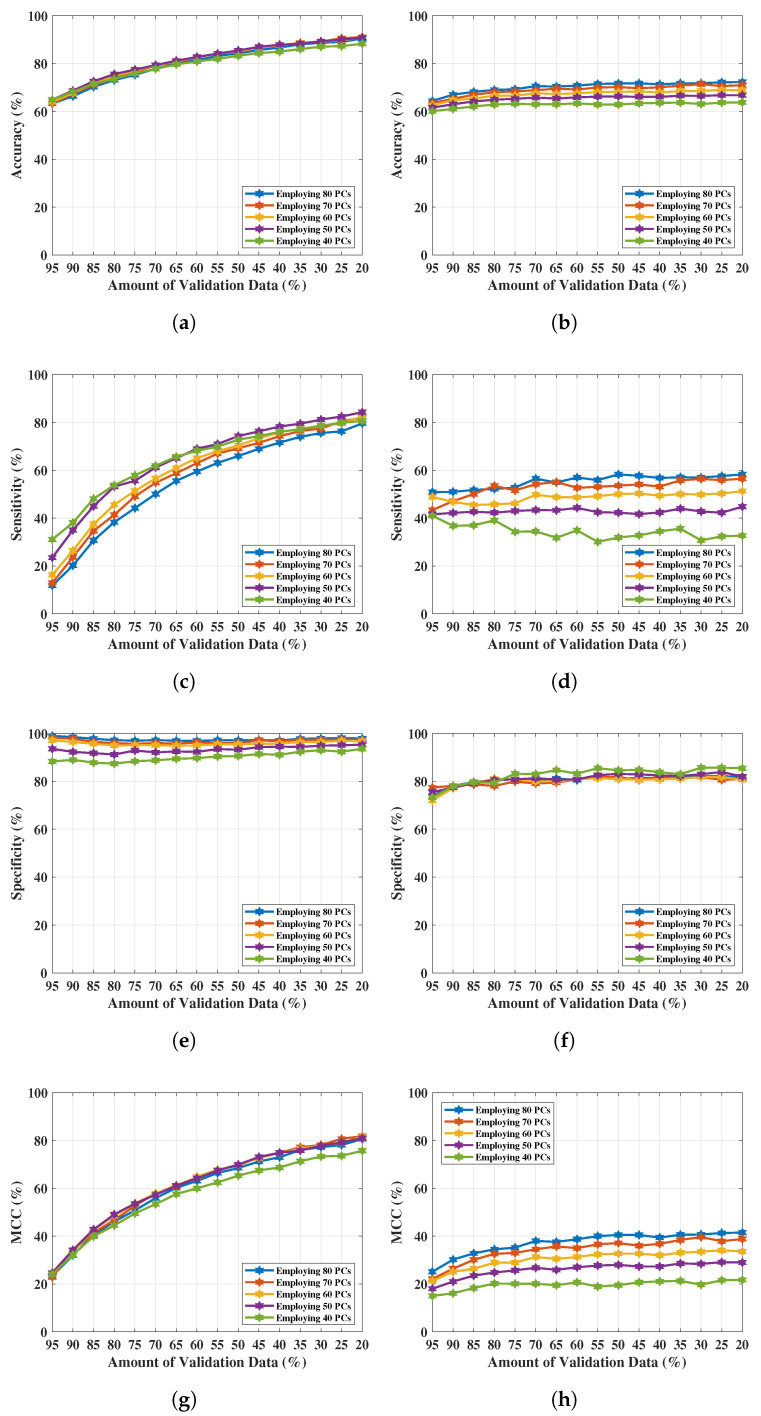
NF and WF signal classification results obtained from $SVM_{Q}$ and $SVM_{G}$ applying PCA over MammoWave’s complex $S_{21}$ data. (**a**) Accuracy obtained from $SVM_{G}$ varying PCs and validation data. (**b**) Accuracy obtained from $SVM_{Q}$ varying PCs and validation data. (**c**) Sensitivity obtained from $SVM_{G}$ varying PCs and validation data. (**d**) Sensitivity obtained from $SVM_{Q}$ varying PCs and validation data. (**e**) Specificity obtained from $SVM_{G}$ varying PCs and validation data. (**f**) Specificity obtained from $SVM_{Q}$ varying PCs and validation data. (**g**) MCC obtained from $SVM_{G}$ varying PCs and validation data. (**h**) MCC obtained from $SVM_{Q}$ varying PCs and validation data.

The results obtained here are from the selection of 80 PCs based on the investigation of Figure 4. With the PC length varied from 80 to 40 at 10-unit intervals, the variation of the classification performance of $SVM_{Q}$ and $SVM_{G}$ was obtained and tabulated in Table 5. Accuracy ($A_{c}$), sensitivity ($S_{e}$), specificity ($S_{p}$), and the Matthews Correlation Coefficient (MCC) performance measure have been computed to investigate the predictions provided by $SVM_{Q}$ and $SVM_{G}$. Table 5a,b show the classification performance of $SVM_{Q}$ and $SVM_{G}$ respectively. Each set of performance metrics varying principal components (PCs) have been included here. The optimal performance of both $SVM_{Q}$ and $SVM_{G}$ in this case was obtained in the first 50 PCs. Outcomes have been plotted in Figure 5 for comparing the performance in graph. Figure 5a,c,e,g show the $A_{c}$, $S_{e}$, $S_{p}$, and MCC respectively obtained from classification applying $SVM_{Q}$. The best sensitivity $S_{e}$ of 0.448 obtained by $SVM_{Q}$ employing 50 PCs which is not significant and satisfactory for breast lesion identification. Though, achieved the specificity $S_{p}$ is 0.820 but the misclassification of breast WF signals (signals reflected from lesions) i.e., false positives lowered the overall $A_{c}$ and MCC. The best set of performance of $SVM_{Q}$ achieved employing 80% of training data (and 20% validation data) with 50 PCs, where $A_{c}=66.80\%$, $S_{e}=44.80\%$, $S_{p}=82\%$, and MCC=29%.

Figure 5b,d,f,h exhibit $A_{c}$, $S_{e}$, $S_{p}$, and MCC respectively by implementing $SVM_{G}$. In case of $SVM_{G}$, all the achieved performance metrics are satisfactory. The best performance of $SVM_{G}$ obtained using 80% of training data (i.e., 20% of validation data) with 50 number of PCs, obtained $A_{c}=90.90\%$, $S_{e}=84.30\%$, $S_{p}=95.30\%$, and MCC=81%. Further reduction of PCs length (40 PCs) drops the performance of classification using $SVM_{G}$ (shown in Figure 5 and Table 5b).

### 4.2. Classification Applying Real parts of Complex $S_{21}$

During the previously executed experiments SVM performed well with the Gaussian kernel, thus the $SVM_{G}$ has been considered here to obtain optimal outcomes for NF and WF breast signal classification. Only real components $\Sigma_{n=1}^{NF}\lambda_{real}\left(n\right)$ from the complex $S_{21}$ signals $\lambda_{n}=\Sigma_{n=1}^{NF=1601}\lambda_{real}\left(n\right)+j\lambda_{img}\left(n\right)$, NF=1601, have been chosen for this classification employing $SVM_{G}$ and the work flow stated in Figure 3. The resistance components $\lambda_{real}\left(n\right)$ have employed as features to inspect whether these are more revealing features for NF-WF signal prediction along with the $SVM_{G}$ and increasing true predications.

$\lambda_{real}\left(n\right)$ data has been analysed and studied through two sample *t*-test for comparing the average values of $\lambda_{real}\left(n\right)$ from two different classes i.e., NF and WF signal data. Table 6 shows the outcomes of the *t*-test, where p<α rejects the null hypothesis $H_{0}$ ($H_{0}=1$), accepts alternative hypothesis $H_{a}$, and the true mean of the population belong between $-6.600\times 10^{-5}$ to $-4.600\times 10^{-5}$. Hence, the acceptance of alternative hypothesis indicates that the ${Real_{S}}_{21}$ data comes from populations with unequal means and can be employed for NF-WF signal classification task.

**Figure 6:**
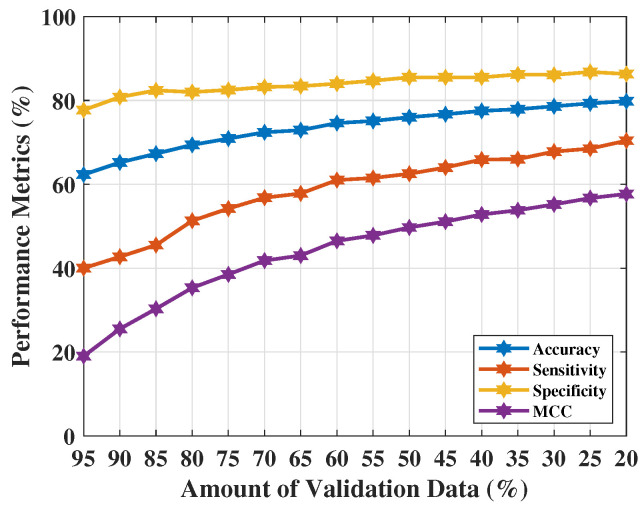
NF and WF signal prediction results (accuracy, sensitivity, specificity, and MCC) obtained using real parts of MammoWave’s complex $S_{21}$ signals applying $SVM_{G}$ over different amount of validation data.

Table 7 shows the NF-WF signal classification performance using $\lambda_{real}\left(n\right)$ applying $SVM_{G}$. The best classification performance of $SVM_{G}$ was obtained with 80% training data (i.e., 20% of validation data). Achieved metrics $A_{c}$, $S_{e}$, $S_{p}$, and MCC are 0.798, 0.704, 0.863, and 0.577 respectively. The performance metrics did not improve on the previous test (using PCs obtained from the actual complex $S_{21}$ sequences). Sensitivity $S_{e}=0.704$ clearly indicates the increment of false positive predictions or misidentification of WF (or, lesion) signals. These misidentifications affect the MCC measure (=0.577) in the other way. All these results have been pictured in Figure 6. This parameter setting becomes unreliable as a significant number of misidentifications were found, also reflected in the performance metrics. The 20% validation data indicates 14,640 number of signals out of which approximately 3597 signals misidentified (false positive signals 2962 approx. and false negative signals 635 approx.) in each run.

### 4.3. Classification Applying PCs of Real Parts of Complex $S_{21}$

**Figure 7:**
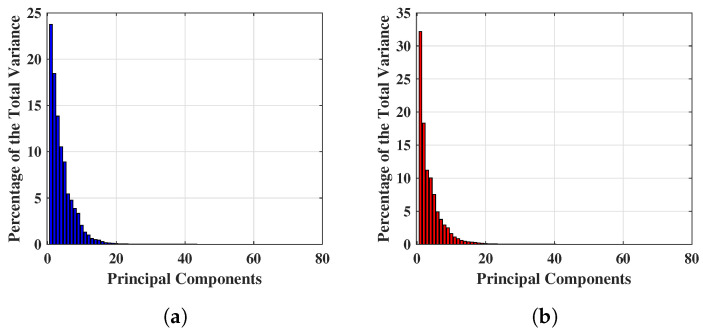
Example of significant variance achieved applying PCA on real part of complex $S_{21}$ signals. (**a**) Significant variance achieved applying PCA on real part of complex $S_{21}$ signals of a NF breast. (**b**) Significant variance achieved applying PCA on real part of complex $S_{21}$ signals of a WF breast.

The third phase of the proposed work has been performed by extracting principal components from $\lambda_{real}\left(n\right)$ and applying $SVM_{G}$ for NF-WF classification (as stated in Figure 3). PCs extracted from $\lambda_{real}\left(n\right)$ are denoted as $\sigma_{1}$, $\sigma_{2}$, …, $\sigma_{n}$. Two vectors of variances (applying PC) have been selected from NF-WF breasts to study the magnitude of variances which has been employed to choose the number of PCs for classification task, as shown in Figure 7. Significant variance has been found upto 80 PCs ($\sigma_{1}$, $\sigma_{2}$, …, $\sigma_{80}$) and selected for NF-WF signal classification. Number of PCs have been varied anticipating an improved performance. In addition, the spherical data distribution and more compactness than before may help in better classification.

The variance of PCs are close to each other in Figure 7. Two sample t-test has been repeated on PCs to understand the capability to represent two signal groups and the data compactness. The probability has been found to be less than the significance level, P<α. Hence, the t-test accepts alternative hypothesis $H_{a}$ and clearly demonstrates the presence of two different means for two different populations. Subsequently, the difference between lower and upper boundary ($-1.770\times 10^{-4}$ and $-1.570\times 10^{-4}$) is reduced which implies improved data compactness than before detailed in Table 8.

**Figure 8:**
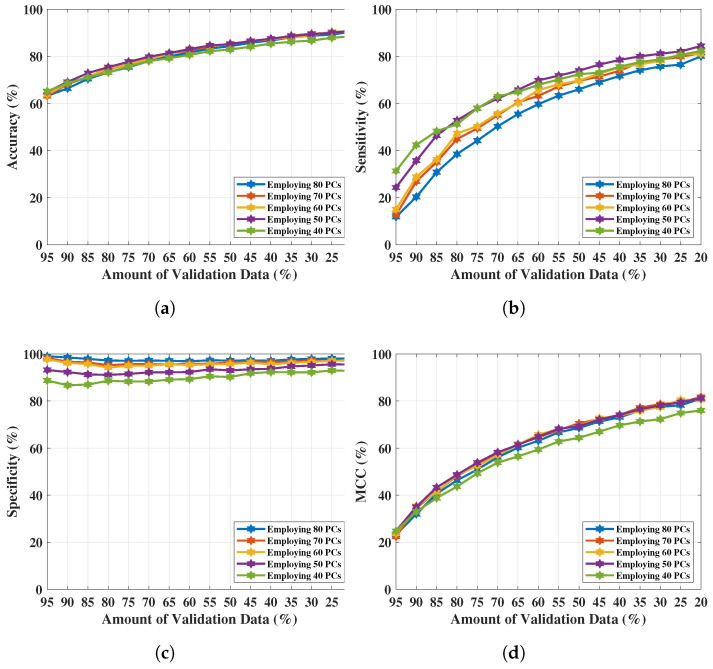
NF and WF signal classification results obtained from $SVM_{G}$ applying PCA over real-parts of MammoWave’s complex $S_{21}$ data. (**a**) Accuracy obtained from $SVM_{G}$ varying PCs and validation data. (**b**) Sensitivity obtained from $SVM_{G}$ varying PCs and validation data. (**c**) Specificity obtained from $SVM_{G}$ varying PCs and validation data. (**d**) MCC obtained from $SVM_{G}$ varying PCs and validation data.

**Figure 9:**
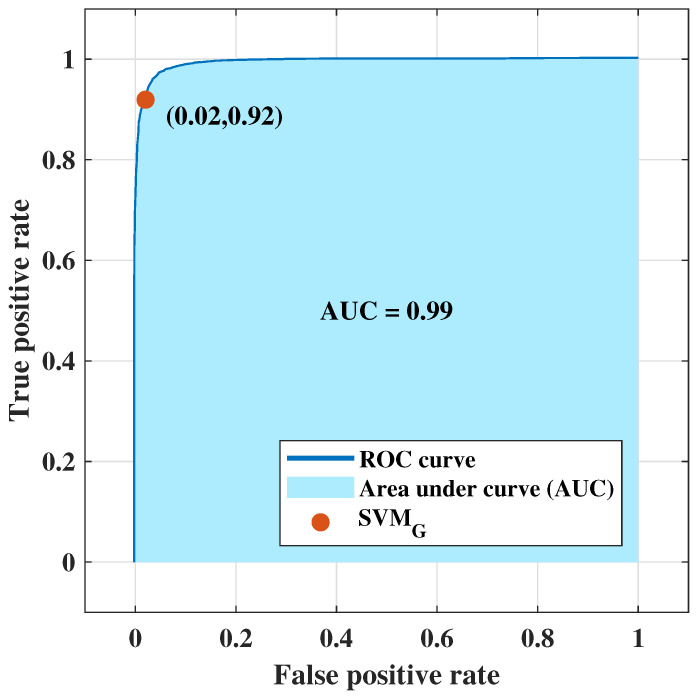
ROC curve obtained from $SVM_{G}$ employing principal components of real parts of $S_{21}$ signals for NF-WF signal classification.

NF-WF signal classification has been performed with the PCs extracted from $\lambda_{real}\left(n\right)$ (up to 80 PCs) and executing $SVM_{G}$ for breast lesion identification. Classification results have been tabulated in Table 9, where $A_{c}$, $S_{e}$, $S_{p}$, and MCC are presented with varying number of PCs (interval of 10 units). The optimal classification performance was achieved with 50 PCs, but begins to decrease upon further reduction (i.e., 40 PCs). The resultant metrics are plotted in Figure 8a–d for comparison between each metric with the PCs variation. Metrics, $A_{c}$, $S_{e}$, $S_{p}$, and MCC increased from 90.90% to 91%, 84.30% to 84.40%, 95.30% to 95.50%, and 81% to 81.20% respectively using the PCs ($\sigma_{1}$, $\sigma_{2}$,…, $\sigma_{50}$) extracted from $\lambda_{real}\left(n\right)$ instead of the PCs extracted from $\lambda_{n}=\Sigma_{n=1}^{NF=1601}\lambda_{real}\left(n\right)+j\lambda_{img}\left(n\right)$. Classification performance improved from the performance achieved in Section 4.1, signifying that PCs derived from resistance components are more enlightening than PCs derived from both $S_{21}$ resistance and reactance components to represent NF-WF breast signals (as well as the NF-WF breasts). Hence, the performance obtained from 50 PCs with $SVM_{G}$ is considered as the optimal validation classification performance. Further two parameters; regularisation and scaling parameter have been tuned to search the space of possible hyperparameter values that results optimum validation results of the proposed model. The regularisation parameter 1 found to be optimal while the model is noticed to be reactive to the variation of kernel scale. Therefore, the ROC curve has been calculated varying kernel scaling parameter to select optimal value, displayed in Figure 9 and measured the area under the ROC curve (AUC = 0.99). The x and y-axes of Figure 9 represents false positive rate (FPR) or (1-specificity) and true positive rate (TPR) or sensitivity respectively. The threshold of ROC curve has been found to be kernel scaling parameter 1.8 for locating the balanced true and false positive rates. Hence, the optimal results $A_{c}$ = 95.07%, $S_{e}$ = 92.40%, $S_{p}$ = 98.32%, and MCC = 89.90% for validation performance have been decided considering kernel scaling parameter 1.8.

### 4.4. Classification Applying Optimal Settings: Training, Validation, Testing Experiment

Once the training and validation process have been completed that apprise the 50 PCs extracted from real parts of complex $S_{21}$ are most efficient for classifying NF and WF signals among all the feature extraction combination employed before. The experiment has now reorganised with the data broken into training, validation, and testing set for addressing data contamination and realise the final unbiased model performance on truly unseen test data set. Table 10 demonstrates the final performance obtained in the proposed work. As limited number of breasts (61 breasts’ data) are available at this stage of research, 20% (12 randomly selected breasts), 25% (15 randomly selected breasts) data have been stratified and held-out using MCCV procedure as test set initially. Rest of the 80%, 75% data have been used for training-validation process using optimal number of 50 PCs of real parts and applying $SVM_{G}$ on 80% allocated training and 20% validation, as settled in Section 4.3. Same performance metrics accuracy, sensitivity, specificity, and MCC have been calculated to analyse the performance. It is found that, the trained and validated $SVM_{G}$ model with 50 PCs of real parts of complex $S_{21}$ outperforms in 20% testing dataset (and 80% training-validation set). The attained performance includes the accuracy $A_{c}$ = 95.50%, sensitivity $S_{e}$ = 97.20%, specificity $S_{p}$ = 94.50%, and MCC = 90.90%, whereas the metrics were $A_{c}$ = 95.00%, $S_{e}$ = 92.40%, $S_{p}$ = 98.30%, and MCC = 89.90% before.

## 5. Discussion & Conclusions

The proposed methodology have three primary focuses: (1) the absence of radiation exposure through MammoWave have many potential benefits compared to the mammography; specifically, women will benefit from safe and accessible radiation-free breast cancer screening, more inclusive (no age-limitation), and more comfortable (no breast compression), (2) the study is part of a retrospective clinical trials, (3) the embrace of robust machine learning models using microwave breast imaging, makes it a cutting edge solution for safe breast lesions detection.

The structure of proposed support vector machine is simple to interpret and performed flexibly with the data. Proposed research outperformed with statistically and biologically dependent data as signals are generated and transmitted using same frequency band for each breast scanning and each patient’s body responds differently to microwave signal transmission. The signal data are fused while preparing for ML application. The procedure is computationally less complex and fast. Results are cross validated using MCCV method. Therefore, the proposed research is protected from several limitations. The experiment in [39] showed that breast frequency response is discriminative and independent where quadratic kernel of SVM can differentiate the signal response reflected from NF and WF breasts with acceptable sensitivity and specificity. However, in [39], $SVM_{Q}$ was applied on a limited number of patients after performing a dedicated $S_{21}$ pre-processing for artefact removal. The proposed work aims to provide a quantitative portrayal of NF-WF breast classification through MammoWave applying the ML algorithm directly to the raw $S_{21}$, i.e., raw-data. In this scenario, it has been found that $SVM_{G}$ outperformed $SVM_{Q}$. Specifically, $SVM_{G}$ was executed on the NF-WF signals of 61 breasts from 35 patients who participated in the feasibility clinical trial, showing satisfactory and improved prediction performance. Accuracy > 91%, sensitivity > 84%, specificity > 95%, and MCC > 81% were achieved through this study. Hence, this proposed study attained remarkable performance in the task of identifying or separating NF and WF (or lesion) signals automatically from raw signal data. The achieved sensitivity (84.40%) and specificity (95.50%) are similar to digital mammography sensitivity [44,45]. However, the MammoWave breast screening is non-invasive and painless and can be used across all ages, during pregnancy and multiple times.

The sensitivity value aligns with the MARIA system which has been used in symptomatic patients [24,25]. The MARIA system (Micrima Ltd, UK) uses an array of 60 antennas and a matching liquid to carry out the radar approach), which is far more complex that the one presented here. The proposed classification algorithm predicted false negatives (actually WF but predicted as NF) in some cases, effecting the sensitivity (84.40%) measure. This was due to the presence of very small sized lesion (few mm or smaller). There are low differences found between the signals value of NF and WF breasts with very small sized lesions. Therefore, numerically NF breasts and WF breasts with very small sized lesion behave similarly, misguiding the classification process, resulting in false negative predictions. This issue will be addressed in our future work by modifying the conventional $SVM_{G}$ kernel structure and performing advance research on feature representation. Moreover, we will investigate the use of microwave image features [22] for dedicated machine learning models, following a procedure similar to [46], where the authors used radiomics derived from Contrast-Enhanced Spectral Mammography Images, obtaining a sensitivity and specificity of 88.37% and 100%.

Raw scan of each breast contains 1200 complex $S_{21}$ signals. Classification of NF-WF signals with high specificity and sensitivity will help to decide the threshold for a breast to be entitled as NF or WF which will help to determine further clinical procedure for the patients. The research for detecting the threshold is underway. Also, further research and more breast data are required to generalise that threshold for breast classification because, a main limitation of the study is represented by the fact that a limited number of breast has been used. Research is in progress using data collected in other MammoWave clinical trials, just ended (https://clinicaltrials.gov/ct2/show/NCT04253366). In this circumstance, ML study will be carried on with ongoing MammoWave clinical trial data [47]. Moreover, further clinical trials are planned to enlarge the resulting research database [48], paving the way for the use of microwave imaging into clinical practice as complementary tool for the screening of asymptomatic women of any age and without any safety restrictions. It is worthwhile pointing out that one of the goals of HORIZON-MISS-2021-CANCER-02-01 scheme is to validate new methods and technologies for cancer screening and early detection, preferably non-invasive and more-inclusive than current approaches. In this context, one of the selected projects has the aim of generating evidence on thousands of women regarding the use of MammoWave as breast cancer screening technique [48].

## Figures and Tables

**Table 1:** Summary of the patient population used in this study.

Name of Parameters	Values
Total patients	34
Total subjects (breasts)	61
Number of patients age between 20–49 year	23
Number of patients age between 50–80 year	38
Mean of patient’s age (in year)	52
Standard deviation of patient’s age (in year)	12

**Table 2:** Details and related radiologist’s review for the breasts with radiological findings (WF).

Age	Breast (L/R)	ACR Breast Density	Mammography BI-RADS	Echography BI-RADS	Radiologist’s Output Details: Sizes (mm) & Notes (if Available)	Pathology or 1-Year Clinical Follow-up Output
48	L	D	3	-	Microcalcifications	Benign
65	L	C	4	-	Cluster of microcalcifications	Benign
40	L	B	2	2	Three masses: 15 mm, 21 mm, and 23 mm	Benign
	R	B	2	2	Microcalcifications	Not available
52	L	C	5	-	Microcalcifications	Malignant
47	L	D	2	2	Microcalcifications	Benign
55	R	C	2	2	1.6 mm microcalcifications	Benign
	L	C	2	2	3.8 mm microcalcifications	Benign
51	L	C	2	2	Presence of metallic marker	Benign
54	R	A	2	2	Microcalcifications	Benign
77	R	D	-	5	17 mm mass	Malignant
61	R	C	4	-	Multifocal lobular type suspected carcinoma (MRI BI-RADS 4)	Malignant
	L	C	2	-	Macrocalcification and Focal contrast enh. (MRI BI-RADS 3)	Not available
50	L	B	2	2	10 mm mass	Benign
67	L	C	4	-	Microcalcifications	Malignant
49	L	A	3	-	Microcalcifications	Benign
70	L	D	3	4	Mass	Malignant
42	L	C	2	3	7 mm mass, hypoechoic	Benign
67	L	B	3	-	Architectural distortion	Benign
56	R	B	4	4	31 mm mass, hypoechoic, irregular borders	Malignant
43	R	D	1	3	12 mm mass	Benign
51	L	C	3	-	Microcalcifications	Benign
59	L	B	-	4	11 mm areolar, suspicious of malignancy	Malignant
40	L	D	2	2	30 mm mass	Benign
35	R	C	2	3	7 mm, hypoechoic	Benign
37	L	A	2	3	25 mm mass	Benign
43	R	B	3	2	Microcalcifications	Malignant
54	R	B	2	2	18 mm mass	Benign
49	L	A	2	3	16 mm mass	Benign
56	L	D	4	4	27 mm mass	Malignant
63	L	A	3	4	6 mm mass	Malignant
55	R	C	4	4	23 mm mass	Malignant
	L	C	2	2	Multiple cysts	Benign
64	R	B	3	-	1.6 mm microcalcifications	Benign
37	R	-	-	3	15.4 mm mass	Benign
	L	-	-	2	Multiple cysts	Not available

**Table 3:** Approximated quantitative summary of raw $S_{21}$ signals, as these values may vary with the addition of new patients.

Breast Type	Mean	Maximum	Minimum	Median	Standard Deviation	Variance
No-Finding (NF)	2.179 × 10${}^{-5}$	0.114	−0.105	−8.286 × 10${}^{-5}$	0.011	4.578 × 10${}^{-7}$
With-Finding (WF)	1.985 × 10${}^{-5}$	0.118	−0.108	−1.640 × 10${}^{-4}$	0.011	4.651 × 10${}^{-7}$

**Table 4:** Two-sample *t*-test on PCA features extracted from MammoWave’s complex $S_{21}$ data.

Null Hypothesis ($H_{\mathrm{null}}$)	Probabilty (*p*)	Confidence Interval ($C_{L}$)	Confidence Interval ($C_{U}$)
1	$7.516\times 10^{-10}$	$-3.000\times 10^{-5}$	$-1.500\times 10^{-5}$

**Table 5:** The classification results over reduced feature space (i.e., extracted features from MammoWave’s original frequency response) after applying SVMs. (a) The classification results over reduced feature space (i.e., extracted features from MammoWave’s original frequency response) after applying $SVM_{Q}$. (b) The classification results over reduced feature space (i.e., extracted features from MammoWave’s original frequency response) after applying $SVM_{G}$.

Feature Dimension →	PC-80				PC-70				PC-60				PC-50				PC-40			
**Validation** **Data**↓	$A_{c}$	$S_{e}$	$S_{p}$	MCC	$A_{c}$	$S_{e}$	$S_{p}$	MCC	$A_{c}$	$S_{e}$	$S_{p}$	MCC	$A_{c}$	$S_{e}$	$S_{p}$	MCC	$A_{c}$	$S_{e}$	$S_{p}$	MCC
95%	0.644	0.509	0.737	0.251	0.635	0.434	0.774	0.221	0.626	0.489	0.720	0.213	0.616	0.416	0.754	0.180	0.601	0.410	0.733	0.150
90%	0.670	0.510	0.781	0.302	0.653	0.471	0.781	0.264	0.647	0.468	0.772	0.251	0.630	0.422	0.775	0.210	0.611	0.368	0.779	0.161
85%	0.682	0.518	0.797	0.328	0.670	0.501	0.787	0.301	0.654	0.455	0.793	0.264	0.642	0.427	0.791	0.235	0.621	0.370	0.796	0.183
80%	0.690	0.522	0.807	0.345	0.680	0.535	0.781	0.326	0.666	0.458	0.811	0.289	0.649	0.423	0.806	0.248	0.629	0.391	0.794	0.202
75%	0.693	0.528	0.808	0.352	0.684	0.516	0.799	0.330	0.666	0.462	0.808	0.289	0.653	0.430	0.808	0.257	0.632	0.343	0.832	0.201
70%	0.706	0.565	0.804	0.380	0.689	0.542	0.791	0.345	0.676	0.498	0.799	0.313	0.658	0.434	0.813	0.268	0.630	0.345	0.830	0.201
65%	0.704	0.549	0.812	0.376	0.695	0.551	0.795	0.357	0.673	0.488	0.801	0.305	0.654	0.433	0.807	0.259	0.630	0.318	0.847	0.195
60%	0.708	0.570	0.805	0.387	0.692	0.527	0.808	0.350	0.676	0.487	0.808	0.313	0.659	0.443	0.808	0.270	0.634	0.349	0.832	0.207
55%	0.715	0.559	0.824	0.400	0.700	0.531	0.818	0.366	0.681	0.492	0.813	0.324	0.662	0.425	0.826	0.277	0.629	0.301	0.855	0.189
50%	0.717	0.583	0.809	0.405	0.702	0.536	0.818	0.371	0.682	0.501	0.809	0.327	0.663	0.423	0.831	0.280	0.629	0.319	0.846	0.195
45%	0.718	0.577	0.815	0.405	0.697	0.541	0.805	0.360	0.683	0.503	0.807	0.327	0.661	0.417	0.829	0.273	0.634	0.328	0.848	0.207
40%	0.713	0.568	0.813	0.394	0.701	0.532	0.819	0.368	0.680	0.494	0.808	0.320	0.661	0.424	0.825	0.273	0.636	0.345	0.838	0.211
35%	0.718	0.571	0.820	0.406	0.708	0.558	0.811	0.384	0.685	0.501	0.812	0.331	0.666	0.440	0.822	0.286	0.637	0.356	0.830	0.213
30%	0.718	0.570	0.822	0.407	0.714	0.564	0.818	0.396	0.686	0.499	0.817	0.335	0.665	0.428	0.830	0.284	0.631	0.307	0.857	0.197
25%	0.722	0.576	0.822	0.413	0.706	0.559	0.806	0.379	0.690	0.503	0.818	0.341	0.668	0.423	0.839	0.291	0.637	0.324	0.857	0.216
20%	0.723	0.583	0.818	0.415	0.710	0.565	0.810	0.388	0.688	0.513	0.807	0.336	0.668	0.448	0.820	0.290	0.638	0.327	0.855	0.217
95%	0.633	0.118	0.990	0.233	0.633	0.129	0.983	0.228	0.641	0.163	0.972	0.242	0.649	0.235	0.936	0.247	0.649	0.311	0.883	0.241
90%	0.664	0.202	0.985	0.320	0.674	0.237	0.978	0.338	0.677	0.264	0.964	0.334	0.687	0.350	0.923	0.342	0.682	0.381	0.890	0.322
85%	0.703	0.307	0.978	0.405	0.711	0.347	0.965	0.414	0.719	0.375	0.957	0.426	0.726	0.450	0.918	0.428	0.716	0.481	0.878	0.399
80%	0.731	0.383	0.972	0.461	0.736	0.414	0.960	0.467	0.748	0.456	0.950	0.485	0.756	0.532	0.912	0.491	0.736	0.538	0.874	0.445
75%	0.753	0.442	0.970	0.508	0.766	0.491	0.957	0.527	0.771	0.514	0.951	0.536	0.775	0.556	0.929	0.536	0.759	0.579	0.884	0.495
70%	0.779	0.501	0.972	0.559	0.792	0.548	0.960	0.579	0.793	0.566	0.951	0.579	0.794	0.612	0.921	0.573	0.778	0.619	0.888	0.534
65%	0.800	0.556	0.970	0.602	0.807	0.588	0.958	0.608	0.810	0.610	0.949	0.612	0.812	0.651	0.925	0.611	0.797	0.657	0.894	0.576
60%	0.815	0.595	0.969	0.631	0.826	0.630	0.962	0.648	0.827	0.651	0.950	0.647	0.828	0.690	0.923	0.642	0.809	0.682	0.897	0.600
55%	0.832	0.631	0.972	0.664	0.842	0.670	0.960	0.678	0.842	0.680	0.955	0.678	0.842	0.710	0.935	0.674	0.820	0.699	0.904	0.625
50%	0.843	0.660	0.971	0.685	0.851	0.692	0.960	0.695	0.852	0.704	0.955	0.698	0.855	0.743	0.932	0.699	0.833	0.728	0.906	0.653
45%	0.857	0.690	0.973	0.712	0.866	0.715	0.971	0.729	0.866	0.734	0.957	0.726	0.870	0.763	0.943	0.731	0.844	0.743	0.914	0.675
40%	0.867	0.716	0.971	0.730	0.876	0.743	0.968	0.748	0.877	0.759	0.958	0.747	0.879	0.783	0.945	0.749	0.850	0.761	0.911	0.687
35%	0.881	0.740	0.978	0.760	0.887	0.764	0.974	0.772	0.885	0.773	0.964	0.765	0.883	0.795	0.944	0.758	0.861	0.772	0.924	0.713
30%	0.887	0.756	0.979	0.772	0.892	0.775	0.974	0.781	0.892	0.787	0.965	0.779	0.893	0.812	0.949	0.778	0.871	0.786	0.930	0.733
25%	0.891	0.763	0.981	0.781	0.906	0.804	0.975	0.807	0.897	0.795	0.969	0.790	0.900	0.825	0.951	0.792	0.873	0.799	0.924	0.736
20%	0.905	0.796	0.979	0.806	0.911	0.820	0.974	0.818	0.910	0.826	0.968	0.815	0.909	0.843	0.953	0.810	0.883	0.806	0.936	0.757

**Table 6:** Two-sample *t*-test on real-parts of MammoWave’s $S_{21}$ data.

Null Hypothesis ($H_{0}$)	Probabilty(*p*)	Confidence Interval ($C_{L}$)	Confidence Interval ($C_{U}$)
1	$8.864\times 10^{-27}$	$-6.600\times 10^{-5}$	$-4.600\times 10^{-5}$

**Table 7:** NF and WF signal classification results obtained from $SVM_{G}$ applying real-parts of MammoWave’s complex $S_{21}$ data.

Validation Data	$A_{c}$	$S_{e}$	$S_{p}$	MCC
95%	0.623	0.400	0.777	0.190
90%	0.652	0.427	0.808	0.255
85%	0.673	0.455	0.824	0.303
80%	0.694	0.513	0.820	0.353
75%	0.709	0.542	0.825	0.385
70%	0.724	0.568	0.832	0.418
65%	0.729	0.578	0.834	0.430
60%	0.746	0.610	0.840	0.465
55%	0.751	0.615	0.847	0.478
50%	0.760	0.625	0.855	0.497
45%	0.767	0.640	0.855	0.511
40%	0.775	0.659	0.855	0.528
35%	0.779	0.660	0.862	0.538
30%	0.786	0.678	0.861	0.552
25%	0.793	0.685	0.868	0.567
20%	0.798	0.704	0.863	0.577

**Table 8:** Two-sample t-test on PCA features extracted from real-parts of MammoWave’s $S_{21}$ data.

Null Hypothesis ($H_{0}$)	Probabilty (*p*)	Confidence Interval ($C_{L}$)	Confidence Interval ($C_{U}$)
1	$8.219\times 10^{-23}$	$-1.770\times 10^{-4}$	$-1.570\times 10^{-4}$

**Table 9:** Classification results applying PCs extracted from real parts of MammoWave’s complex $S_{21}$ signals with $SVM_{G}$.

Feature Dimension →	PC-80				PC-70				PC-60				PC-50				PC-40			
**Validation** **Data**↓	$A_{c}$	$S_{e}$	$S_{p}$	MCC	$A_{c}$	$S_{e}$	$S_{p}$	MCC	$A_{c}$	$S_{e}$	$S_{p}$	MCC	$A_{c}$	$S_{e}$	$S_{p}$	MCC	$A_{c}$	$S_{e}$	$S_{p}$	MCC
95%	0.632	0.118	0.990	0.232	0.632	0.127	0.983	0.224	0.638	0.150	0.977	0.237	0.650	0.243	0.932	0.248	0.652	0.313	0.887	0.248
90%	0.664	0.203	0.985	0.320	0.682	0.269	0.967	0.346	0.686	0.288	0.962	0.354	0.691	0.357	0.923	0.351	0.686	0.424	0.867	0.330
85%	0.704	0.308	0.979	0.407	0.712	0.351	0.964	0.416	0.712	0.362	0.957	0.413	0.729	0.464	0.913	0.433	0.711	0.482	0.870	0.388
80%	0.732	0.385	0.972	0.463	0.746	0.449	0.952	0.482	0.750	0.473	0.942	0.487	0.754	0.528	0.911	0.487	0.732	0.512	0.886	0.437
75%	0.754	0.442	0.971	0.509	0.767	0.494	0.957	0.529	0.766	0.503	0.950	0.524	0.777	0.580	0.915	0.538	0.758	0.579	0.883	0.493
70%	0.780	0.503	0.972	0.561	0.790	0.550	0.957	0.575	0.788	0.557	0.950	0.570	0.798	0.621	0.922	0.582	0.780	0.630	0.883	0.538
65%	0.800	0.555	0.971	0.602	0.812	0.605	0.955	0.617	0.810	0.601	0.956	0.615	0.814	0.658	0.922	0.614	0.792	0.649	0.891	0.565
60%	0.816	0.597	0.969	0.631	0.825	0.632	0.959	0.645	0.832	0.657	0.953	0.657	0.831	0.698	0.923	0.649	0.806	0.679	0.893	0.594
55%	0.833	0.633	0.973	0.667	0.842	0.672	0.958	0.677	0.844	0.681	0.956	0.681	0.846	0.718	0.935	0.682	0.822	0.702	0.905	0.628
50%	0.843	0.660	0.972	0.686	0.854	0.696	0.964	0.705	0.851	0.697	0.957	0.695	0.852	0.739	0.930	0.693	0.829	0.724	0.902	0.644
45%	0.858	0.690	0.973	0.713	0.864	0.714	0.968	0.724	0.866	0.727	0.963	0.727	0.865	0.765	0.935	0.721	0.841	0.731	0.918	0.670
40%	0.867	0.717	0.971	0.731	0.873	0.739	0.966	0.741	0.873	0.753	0.956	0.739	0.875	0.785	0.937	0.741	0.854	0.756	0.923	0.697
35%	0.881	0.741	0.977	0.760	0.888	0.775	0.967	0.772	0.881	0.766	0.962	0.758	0.886	0.800	0.947	0.765	0.862	0.776	0.922	0.713
30%	0.888	0.757	0.979	0.775	0.896	0.785	0.972	0.787	0.890	0.781	0.966	0.776	0.894	0.811	0.951	0.781	0.867	0.787	0.922	0.723
25%	0.892	0.765	0.981	0.782	0.900	0.798	0.970	0.795	0.905	0.809	0.971	0.805	0.900	0.821	0.956	0.795	0.879	0.806	0.930	0.749
20%	0.906	0.800	0.980	0.810	0.910	0.813	0.977	0.817	0.905	0.810	0.972	0.806	0.910	0.844	0.955	0.812	0.885	0.823	0.928	0.760

**Table 10:** Training, validation, and testing dataset: classification results applying PCs extracted from real parts of MammoWave’s complex $S_{21}$ signals with $SVM_{G}$.

Total Breasts	Training-Validation Data	Training Data	Validation Data	Feature Dimension PC-50				Testing Data	Feature Dimension PC-50			
				$A_{c}$	$S_{e}$	$S_{p}$	MCC		$A_{c}$	$S_{e}$	$S_{p}$	MCC
61 Breasts	75% of Data (46 Breasts)	80%	20%	84.20%	88.20%	82.20%	67.40%	25% of Data (15 breasts)	94.40%	96.20%	93.40%	88.50%
61 Breasts	80% of Data (49 Breasts)	80%	20%	85.40%	88.80%	83.60%	69.70%	20% of Data (12 breasts)	95.50%	97.20%	94.50%	90.90%

## Data Availability

The datasets that support the findings of this study are not publicly available, but will be made available upon reasonable request, following ethics committee approval and a data transfer agreement to guarantee the General Data Protection Regulation. Please contact the authors, Soumya Prakash Rana (Email: ranas11@lsbu.ac.uk, soumyaprakash.rana@gmail.com) or Gianluigi Tiberi (Email: tiberig@lsbu.ac.uk, gianluigi@ubt-tech.com) to request access to the data.
